# Assessment of Menthol and Nonmenthol Cigarette Consumption in the US, 2000 to 2018

**DOI:** 10.1001/jamanetworkopen.2020.13601

**Published:** 2020-08-07

**Authors:** Cristine D. Delnevo, Daniel P. Giovenco, Andrea C. Villanti

**Affiliations:** 1 Rutgers Center for Tobacco Studies, New Brunswick, New Jersey; 2 Department of Health Behavior, Society and Policy, Rutgers School of Public Health, Piscataway, New Jersey; 3 Department of Sociomedical Sciences, Columbia University Mailman School of Public Health, New York, New York; 4 Vermont Center on Behavior and Health, Department of Psychiatry, University of Vermont, Burlington

## Abstract

This cross-sectional study examines trends in menthol cigarette use compared with nonmenthol cigarette use in the US.

## Introduction

The 2009 Family Smoking Prevention and Tobacco Control Act (TCA) provided the US Food and Drug Administration (FDA) with broad authority to regulate tobacco products. The TCA banned flavors in cigarettes, except menthol, and tasked the FDA with studying menthol cigarettes with an emphasis on specific groups, given high rates of use among youth smokers (52.5%) and Black smokers (86.5%).^1^ The high rates of menthol cigarette use among Black smokers is driven by decades of targeted marketing by the tobacco industry.^1,2^ The FDA’s Tobacco Product Scientific Advisory Committee concluded that menthol in cigarettes reduces the harshness of smoke and is associated with increased initiation,^2,3^ higher dependence, and lower quit success,^2^ particularly among Black smokers.^4^ This study updates previous estimates of menthol and nonmenthol cigarette consumption through 2018.^5^

## Methods

This serial cross-sectional study estimates menthol and nonmenthol cigarette consumption from 2000 to 2018 with a previously used approach.^5^ The Rutgers institutional review board determined that this study met the criteria for non–human subjects research. This study followed the Strengthening the Reporting of Observational Studies in Epidemiology (STROBE) reporting guideline.

First, annual cigarette consumption data were obtained from the Tobacco Tax and Trade Bureau. Second, the menthol market share was estimated by calculating the mean for each year from 3 sources: the Maxwell Report (2000-2014), the US Federal Trade Commission’s cigarette reports (2000-2018), and the Euromonitor data (2004-2018). Third, the annual menthol and nonmenthol cigarette consumption was estimated by multiplying the estimated menthol market share by total cigarette consumption. Descriptive analyses were conducted using SPSS Statistics software, version 26 (IBM) in March 2020.

## Results

As shown in the Table, menthol cigarette market share increased by nearly 10 percentage points from 2000 (mean market share of 25.90% [95% CI, 24.63%-27.17%]) to 2018 (mean market share of 35.40% [95% CI, 29.08%-41.72%]). Overall, cigarette consumption declined 46.0% from 2000 (435.6 billion cigarettes) to 2018 (235.3 billion cigarettes), but the decline was greater among nonmenthol cigarettes (52.9%; 322.8 billion to 152.0 billion cigarettes) than for menthol cigarettes (26.1%; 112.8 billion to 83.3 billion cigarettes in 2018) (Figure). Indeed, 85% of the total decline in cigarette consumption was attributed to nonmenthol cigarettes. Moreover, since the TCA was signed, consumption declined 33.1% for nonmenthol cigarettes (227.0 billion to 152.0 billion cigarettes) but only 8.2% for menthol cigarettes (90.8 billion to 83.3 billion cigarettes), with 91% of the decline between 2009 and 2018 attributed to nonmenthol cigarettes.

**Table.** Estimated Menthol Market Share in the US, 2000-2018^a^

Year	Market share, %			
	FTC	Euromonitor	Maxwell	Mean (95% CI)
2000	26.0	NA	25.8	25.90 (24.63-27.17)
2001	26.0	NA	26.0	26.00 (26.00-26.00)
2002	27.0	NA	26.4	26.70 (22.89-30.51)
2003	27.0	NA	25.6	26.30 (17.41-35.19)
2004	27.0	26.2	24.2	25.80 (22.22-29.38)
2005	27.0	26.4	24.7	26.03 (23.07-29.00)
2006	28.0	26.3	25.5	26.6 (23.43-29.77)
2007	28.9	26.8	28.3	27.99 (25.32-30.67)
2008	26.7	27.0	29.2	27.63 (24.23-31.03)
2009	29.0	27.4	29.3	28.57 (26.03-31.10)
2010	30.8	28.3	30.2	29.76 (26.55-32.97)
2011	32.4	29.2	30.7	30.75 (26.83-34.67)
2012	32.8	29.6	31.1	31.18 (27.15-35.21)
2013	33.5	30.1	31.4	31.68 (27.37-35.99)
2014	33.0	30.5	31.9	31.79 (28.71-34.88)
2015	34.4	30.9	NA	32.63 (10.67-54.58)
2016	35.2	33.7	NA	34.44 (25.07-43.81)
2017	35.5	34.1	NA	34.82 (25.63-44.01)
2018	35.9	34.9	NA	35.40 (29.08-41.72)

Abbreviations: FTC, US Federal Trade Commission; NA, not applicable.

^a^ Menthol market share data not available for Euromonitor prior to 2004; the Maxwell Report ceased producing an estimate of menthol market share in 2015, and ceased their report in 2017.

**Figure.**
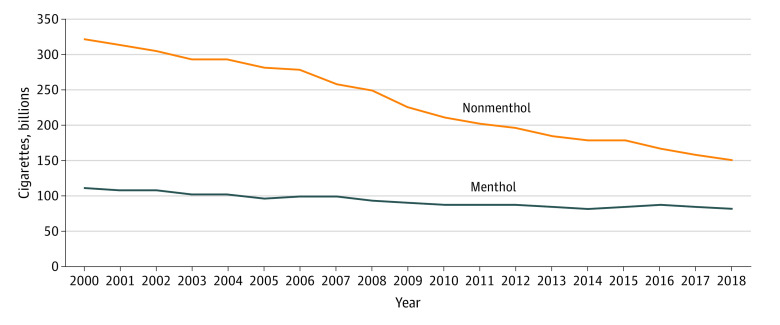
Estimated Menthol and Nonmenthol Cigarette Consumption in the US, 2000-2018

## Discussion

The large decline in cigarette consumption in the US over the last 2 decades is overwhelmingly attributed to nonmenthol cigarettes, and this trend is more pronounced since the signing of the TCA. Comparatively stable consumption of menthol cigarettes is consistent with the Tobacco Product Scientific Advisory Committee’s conclusion that menthol in cigarettes increases regular smoking and results in lower likelihood of cessation.^2^ Of note, there have been changes in the market in the last decade that promote menthol smoking, such as menthol flavor capsules, which appeal to young adults,^6^ and have been gaining market share.

The Tobacco Product Scientific Advisory Committee also concluded that the removal of menthol cigarettes from the marketplace would benefit public health in the US. In 2013 and again in 2018, the FDA issued advance notices of proposed rulemaking specific to menthol in cigarettes, but has yet to act. In 2020, the US House of Representatives passed a bill that included a ban on the sale of menthol cigarettes, but its fate is unclear in the Senate. States and local jurisdictions have the authority to address menthol cigarette sales; pending federal action, these efforts should be prioritized. On June 1, 2020, the first statewide ban on menthol cigarettes took effect in Massachusetts.

This study has limitations. One limitation is that consumption data cannot characterize the epidemiology of menthol smoking. However, the data underscore that menthol in cigarettes may be stalling progress in reducing cigarette smoking, and potentially perpetuates existing health disparities. Policies to ban menthol cigarettes would benefit public health and should not be delayed further.
